# Growth Inhibition and Allelopathy Enhancement of *Alternanthera philoxeroides* Under Long-Term Exposure to Different Sound Intensities

**DOI:** 10.3390/plants15111678

**Published:** 2026-05-29

**Authors:** Ai-Ping Wu, Yu-Han Xiao, Wen-Qi Duan, Le Qiao, Gao-Bin Xiang, Hui Fu, Gui-Xiang Yuan, You-Zhi Li, Yan-Hong Wang, Mohamed Abdelaziz Balah, Jin-Rui Yuan, Chang-Liang Shao

**Affiliations:** 1 Hunan Provincial Key Laboratory of Rural Ecosystem Health in Dongting Lake Area, Ecology Department, College of Environment and Ecology, Hunan Agricultural University, Changsha 410128, China; 2 School of Forestry and Bio-Technology, Zhejiang Agriculture & Forestry University, Hangzhou 311300, China; 3 Plant Protection Department, Ecology and Dry Lands Agriculture Division, Desert Research Center, 1 Mathaf El Matariya Street, El Matariya, Cairo 11753, Egypt; 4 State Key Laboratory of Efficient Utilization of Arid and Semi-Arid Arable Land in Northern China, National Hulunber Grassland Ecosystem Observation and Research Station, Institute of Agricultural Resources and Regional Planning, Chinese Academy of Agricultural Sciences, Beijing 100081, China

**Keywords:** allelopathy, *Alternanthera philoxeroides*, long-term exposure, antioxidant defense, sound rhythm, growth inhibition

## Abstract

Although numerous studies have explored the effects of various sounds on plants, a comparative understanding of how long-term exposure to low-intensity sound and rhythmic versus non-rhythmic sound impact on plants is still lacking. In this study, we conducted a field experiment to determine the growth, physiological responses, and allelopathic intensity of an invasive plant *Alternanthera philoxeroides* under prolonged exposure to different sound rhythms and intensities. The results showed that even at low intensity, long-term sound exposure inhibited the growth of *A. philoxeroides* while activating its defense mechanisms; these responses intensified with increasing sound intensity. However, the plant could not well distinguish between rhythmic and non-rhythmic sound treatments, although its allelopathic intensity also increased with sound intensity. This study extends the knowledge of plant response to acoustic stimuli, highlights the limited ability of plants to discriminate sound rhythms, and underestimates the negative impacts of prolonged low-intensity sound on plant performance. Therefore, the application of acoustic treatment techniques in future horticulture and agriculture requires a careful trade-off between their potential benefits and adverse effects on plants.

## 1. Introduction

Sound is a form of mechanical radiant energy that propagates as longitudinal pressure waves through gas, liquid, or solid material mediums [1], characterized by its wavelength hertz (Hz), intensity (decibel, dB), speed, and direction [2]. Sound is categorized as audible (sonic or ordinary) (20 Hz–20 kHz), infrasonic (<20 Hz), and ultrasonic (>20 kHz) based on human perception. As ubiquitously ambient signals or stimuli, sound waves are known to exert mechanical impacts on both living organisms and inorganic matters they encounter [3]. Although sound shares physical similarities with tactile stimuli, plants can properly distinguish between sound and touch, as well as between relevant and irrelevant acoustic cues [3]. Despite their sessile nature, plants are not passive; they demonstrate active behaviors such as learning, memory, and forms of intelligence, albeit without consciousness [4,5,6,7,8]. Consequently, as highly sensitive organisms, plants constantly encounter diverse sounds, and are capable of perceiving, interpreting, and interacting with acoustic waves even though in the absence of specialized sound-sensory organs [4,8,9,10,11,12].

Plants can perceive sound through direct mechanical contact, vibration, and transmission via soil-, water-, or airborne media [12,13,14], a process facilitated by their touch receptors [15]. Furthermore, plants can spontaneously generate their own sound, typically in the high-frequency range, or release sound emissions from their xylem motivated by some environmental signals [11,16,17]. This acoustic emission is generally produced through a cavitation process [14]. Sound perception has been shown to promote various plant processes, including seed germination, pollination, threat avoidance, stress acclimation, and inter-plant communication [18,19]. A growing body of evidence shows that sound stimulation can induce various changes in plant systems, such as alterations in gene expression, epigenetic modifications, hormone signaling, secondary metabolite production, callus proliferation, photosynthetic efficiency, germination, growth, flowering, defense responses, and disease resistance [4,20,21,22,23,24,25,26]. Additionally, sound can modulate plant stomatal opening and closing, enhancing the assimilation of pesticides or fertilizers, thereby supporting energy conservation and environmental protection through reduced chemical applications [14,27]. Moreover, plants can learn to associate specific sound cues with available resources and encountered stressors, leading to adaptive physiological responses [28,29]. Given these multifaceted effects, acoustic treatment has emerged as a prosperous technique to improve seed germination, plant growth, and crop yield in horticulture and agriculture [29,30,31].

However, plant responses to sound vary depending on wavelength and intensity. In general, sound waves with shorter wavelengths and lower intensities, such as classical music or natural sound, tend to promote plant growth [21,23,24,26,32,33,34]. In contrast, sound waves with longer wavelengths and higher intensities, including loud rock music or noise, often inhibit growth [19,32,33]. This difference is primarily attributed to the greater energy and sound pressure associated with the latter category [21,35]. For instance, paddy rice seed germination is suppressed when exposed to sound waves above 4 kHz or 111 dB [32]. Notably, each plant species may have a specific optimal sound pressure range for growth stimulation. While appropriate sound levels can significantly enhance plant development, exceeding a certain threshold tends to inhibit it [32,34,35]. Typically, the growth-promoting effects of sound pressure are generally linked to elevated levels of phytohormones [10,14], such as indole-3-acetic acid and gibberellin, and increased concentrations of nutrients like soluble sugars and proteins [24,26,35].

However, most previous studies have focused on the effects of sound wavelength and intensity on plants [21,22,23,24,26]; very few have investigated plant responses to sound with different rhythms [19]. Plants may adapt to rhythmic variations resulting from distinct vibrational patterns [19]. In acoustic terms, music consists of rhythmic sound characterized by varying frequencies, vibrations, shapes, qualities, points, and waveforms [17], whereas noise is defined as non-rhythmic, non-harmonic, and undesirable sound, and natural sound encompasses a mixture of rhythmic and non-rhythmic environmental elements, such as environmental cues like wind and bicker as well as biotic signals like animal calls [36]. Accordingly, musical notes possess specific defined frequencies, noise is more closely associated with high sound intensity, and natural sound falls between these two categories [37]. Whether plants can distinguish between different sound rhythms and how they respond to them therefore merits further investigation.

Moreover, most former sound experiments were conducted over limited time periods [5,26,38]. Nevertheless, results indicate that sound can induce notable physiological and structural changes in plants over time, often positive under low-intensity conditions [39], while prolonged exposure to noise (high-intensity sound) can adversely affect plant systems [19]. However, it remains unclear whether plants can be suppressed by long-term exposure to short-wavelength, low-intensity sound, a form of abiotic stress [1,27].

In this study, we conducted a field experiment to determine the growth, physiological responses, and allelopathic intensity of an invasive plant *Alternanthera philoxeroides* (Mart.) Griseb. under different sound rhythm conditions. Despite varying nutrient availability, this species has been shown to significantly inhibit co-occurring native plants through the release of allelochemicals [40,41,42]. We hypothesized that alligator weed could distinguish between musical and non-musical sound rhythms, given that plants respond distinctly to different vibrational sound patterns [19]. Moreover, we expected that prolonged exposure to low-intensity sound would inhibit plant growth while enhancing defensive responses and allelopathic effects, owing to its role as an abiotic stressor [1,27]. Finally, we assumed that plant growth inhibition and physiological excitement as well as allelopathic inhibitory effects in alligator weed would increase with sound intensity because of an increase in sound stress [1,29].

## 2. Materials and Methods

### 2.1. Sound Experiment

#### 2.1.1. Experimental Design

This experiment was conducted from 20 June to 19 October 2023 at the Liuyang Experimental Base of Hunan Agricultural University (28.30° N, 113.82° E), a region characterized by a mid-subtropical monsoon humid climate with a mean annual temperature of 17 °C and mean annual precipitation of 1128.5 mm. During the study period, temperature ranged from 26 °C to 43 °C. A total of 180 robust apical unbranched shoots of *A. philoxeroides* (mean initial weight 5.80 ± 0.76 g (W_0_), length 10 cm and similar morphology) were collected from an abandoned field at the base and used for planting. In every 2 m^2^ circular plot, six plant individuals were evenly planted 5 cm deep at a distance of 50 cm from the plot center. The soil properties were as follows: organic matter 21.63–23.19 g kg^−1^, total nitrogen 0.83–0.95 g kg^−1^, total phosphorus 0.11–0.23 g kg^−1^, and pH 6.15–6.42.

After two weeks of acclimation, the plants were exposed to five different sound treatments: control (CK-S), light music (LM), natural sound (NS), noise sound (NM), and rock music (RM), with six replicates per treatment. Light music and rock music were considered rhythmic sound, while natural sound and noise sound were considered non-rhythmic sound. A speaker (except in CK-S) was hung at the center of each plot, 50 cm above the ground. All sound treatments (LM, NS, NM, and RM) were applied at a frequency of 1200 Hz, with fixed sound pressure levels of 60 dB (LM, NS) and 120 dB (NM, RM), representing low and high sound intensities, respectively. Sound exposure was provided daily from 8:00 a.m. to 10:00 a.m. and these sound treatments lasted for four months. As the duration of each sound clip was only a few minutes, the same clip was repeatedly played during the exposure period, and the audio files are provided as Supplementary Materials. Plots of the same treatment were spaced 1 m apart, whereas different treatments were separated by >120 m to avoid acoustic interference. Weeds (any plants not originally planted) were carefully removed at an early stage, and other necessary maintenance was performed to minimize external disturbances. The experiment employed five sound treatments with six replicates each, resulting in a total of 30 plots.

#### 2.1.2. Parameter Measurements

Four months later, the chlorophyll content of the third newest pair of fully expanded leaves on the longest ramet of each plant was measured using a portable SPAD-502 chlorophyll meter (Konica Minolta Sensing, Inc., Tokyo, Japan). The average value of the six plants in each plot was recorded as the leaf chlorophyll content for that plot. Subsequently, plants were carefully uprooted, gently washed to remove soil, and blotted dry. The fresh mass (W_1_) of each individual was determined, and the mean fresh mass of the six plants in a plot was taken as the plant mass of the plot. The relative growth rate (RGR) for each plot was calculated using the formula [43]:

RGR = (lnW_1_ − lnW_0_)/t

where W_0_ and W_1_ were the initial mass (5.8 g) and final plant fresh mass in the experiment, respectively, and t was the duration of the experiment in days. The RGR value for each plot was derived as the mean value of the six individuals in that plot.

The leaf samples were divided into two portions: one for determining malondialdehyde (MDA) concentration, superoxide dismutase (SOD) activity [44], and preparing aqueous extracts, and the other for quantifying total phenolic content. Frozen samples were ground into a fine powder in liquid nitrogen using a mortar and pestle. For MDA and SOD analysis, 1.0 g of powder was extracted for 20 min at 4 °C in 5 mL of ice-cooled phosphate-buffered saline (PBS, 0.05 M, pH 7.8), followed by centrifugation at 15,000× *g* for 10 min at 4 °C. The resulting supernatant was used for the assays, with MDA concentration and SOD activity measured according to the manufacturer’s instructions (Solarbio Life Sciences, Beijing, China). For total phenol determination, leaves were first dried at 105 °C for 30 min, then oven-dried at 65 °C for 72 h, ground to a fine powder, and passed through a 0.45 μm sieve. Total phenols were subsequently quantified using the Folin–Ciocalteu colorimetric method [45].

### 2.2. Allelopathic Experiment

#### 2.2.1. Experimental Design

After air-drying, plant samples were ground into a fine powder using a mortar and pestle. Aqueous leaf extracts were prepared by suspending 5 g of the powder in 1 L of distilled water at room temperature (25 ± 3 °C) with occasional stirring. After 48 h of extraction, the mixture was filtered through four layers of cheesecloth to remove solid residues. The pH of the resulting aqueous extract was adjusted to 7.0 with 1 M NaOH or HCl, yielding a final concentration of 5 g/L, a commonly used concentration in allelopathy studies [46]. All extracts were stored at 4 °C until use. Seeds of *Lactuca sativa* and *Medicago sativa* were obtained from Xiang Nong You Pin Seed Company (Changsha, China). Prior to the experiment, seeds were surface-sterilized in 1% sodium hypochlorite for 15 min and then rinsed thoroughly with deionized water several times.

Before experiment, a preliminary test confirmed that the allelopathic effects (inhibitory or promotive) observed on the two bioassay species were attributable to allelochemicals in the leaf aqueous extracts of *A. philoxeroides*, rather than to osmotic potential. Accordingly, six treatments were established using leaf aqueous extracts of *A. philoxeroides* from the CK-S, LM, NS, NM, and RM sound-exposure groups, along with a control (CK-W: distilled water without plant material). Two target species, *L. sativa* and *M. sativa*, were used. Each extract treatment was replicated six times for each target species, resulting in a total of 12 treatments (6 extract types × 2 species = 12 treatments). In each treatment, 40 surface-sterilized, uniformly sized, and healthy seeds of the target species were evenly distributed in a Petri dish (diameter: 9 cm) lined with two layers of filter paper, to which 3 mL of the corresponding *A. philoxeroides* aqueous extract or distilled water (CK-W) was added. The covered dishes were then placed in a climate-controlled incubator under a 12 h light (28 °C)/12 h dark (20 °C) photoperiod, with relative humidity maintained above 75% (reflecting the typical climate of Hunan Province). During the germination period, filter papers were moistened as needed with the respective extract or distilled water to prevent drying. Germinated seeds were counted daily. The experiment was terminated on the seventh day when no new germination had occurred for two consecutive days.

#### 2.2.2. Index Measurements

The seeds were considered to be germinated when the radicle had visibly penetrated the seed coat. The germination rate (GR) was calculated as the proportion of germinated seeds to the total number of seeds tested. Germination speed (GS) was determined according to the method of [46], and germination potential (GP) was defined as the proportion of seeds germinated on the third day relative to the total number tested [27]. The germination index (GI) was computed as GI = ∑Gi/Dt, where Gi is the number of normally germinated seeds on day t, and Dt is the corresponding number of days after cultivation [47]. On the fourth day, ten representative seedlings that had germinated between the third and fourth days were randomly selected for measurement of root length (RL) and fresh mass (FM). A vigor index (VI) was then calculated as the arithmetic mean of GI and FM [48]. Finally, a synthetic allelopathic effect index was derived as the arithmetic mean of all measured parameters (GS, GR, GP, GI, VI, RL, and FM) for each recipient plant species.

### 2.3. Data Analysis

To assess the effects of sound treatments on plant mass, SPAD, relative growth rate (RGR), malondialdehyde (MDA), superoxide dismutase (SOD) activity, and total phenols in alligator weed, a one-way ANOVA was performed, followed by Tukey’s post hoc tests. Similarly, a two-way ANOVA was used to evaluate the effects of plant species (*L. sativa* and *M. sativa*) and aqueous extracts (including the distilled-water control) derived from plants exposed to five sound treatments on the eight measured germination-related indices: germination speed (GS), germination rate (GR), germination potential (GP), germination index (GI), vigor index (VI), root length (RL), fresh mass (FM), and synthetic allelopathic effect. Tukey’s post hoc tests were applied for multiple comparisons. All ANOVAs were conducted at the 95% confidence level, and homogeneity of variances was tested using Levene’s test. Based on the sum of squares (SS) from the two-way ANOVA, variance partitioning was performed to quantify the contribution of each source to the total variance in the response variables, following the approach of Yuan et al. [43]. The total SS was decomposed as:

SS_total_ = SS_species_ + SS_sound_ + SS_species × sound_ + SS_error_

Then, the proportional contribution of each source was expressed as a percentage of SS_total_. In this study, all statistical analyses were conducted using the software SPSS 23.0 (IBM Corp., Armonk, NY, USA).

## 3. Results

### 3.1. Effects of Sound on Plant Growth and Antioxidant Defense

**Figure 1:**
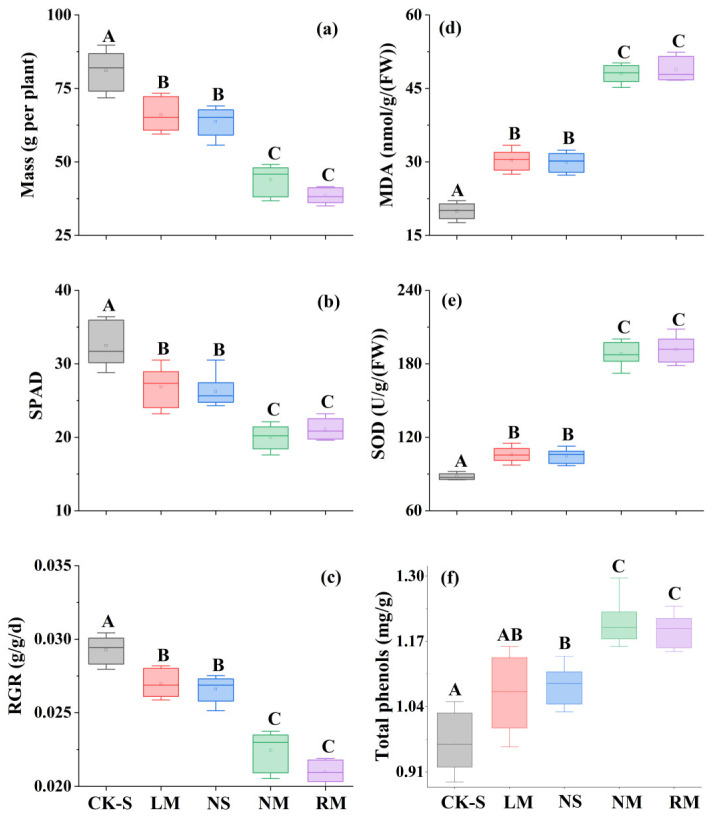
The effects of different sound rhythms on plant mass (**a**), SPAD (**b**), relative growth rate, RGR (**c**), malondialdehyde, MDA (**d**), superoxide dismutase, SOD (**e**), and total phenols (**f**). Note: Bars labeled with different letters represent significantly different (*p* < 0.05; one-way ANOVA), bars represent standard errors; CK-S: control without sound treatment, LM: light music, NS: natural sound, NM: noise sound, and RM: rock music.

The results showed that the plant mass, SPAD, and relative growth rate (RGR) of the invasive alligator weed were significantly suppressed under all four sound treatments compared with the control (Figure 1, *p* < 0.05). Moreover, the magnitude of suppression increased with sound intensity, but no significant difference was observed between rhythmic (music) and non-rhythmic sound at either low or high intensity (Figure 1, *p* > 0.05). In contrast, malondialdehyde (MDA) content, superoxide dismutase (SOD) activity, and total phenol levels were markedly enhanced by all sound treatments relative to the control (Figure 1, *p* < 0.05). These stimulatory effects also increased with sound intensity, but again did not differ significantly between rhythmic and non-rhythmic sound at low or high intensity (Figure 1, *p* > 0.05).

### 3.2. Allelopathic Effects on M. sativa

**Figure 2:**
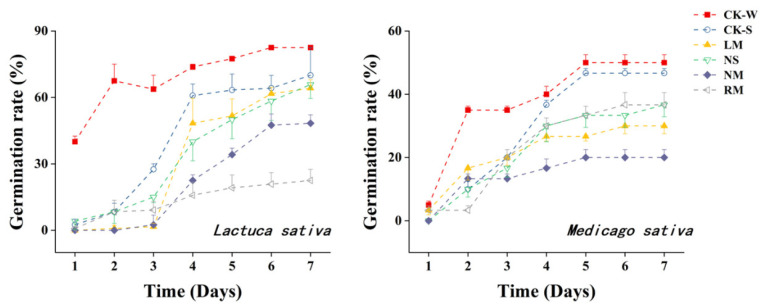
Allelopathic effects of aqueous extracts of *A. Philoxeroides* exerted by different sound treatments on germination rate of two bioassay species. Note: Bars represent standard errors; CK-W: control of distilled water, CK-S: control without sound treatment, LM: light music, NS: natural sound, NM: noise sound and RM: rock music.

**Figure 3:**
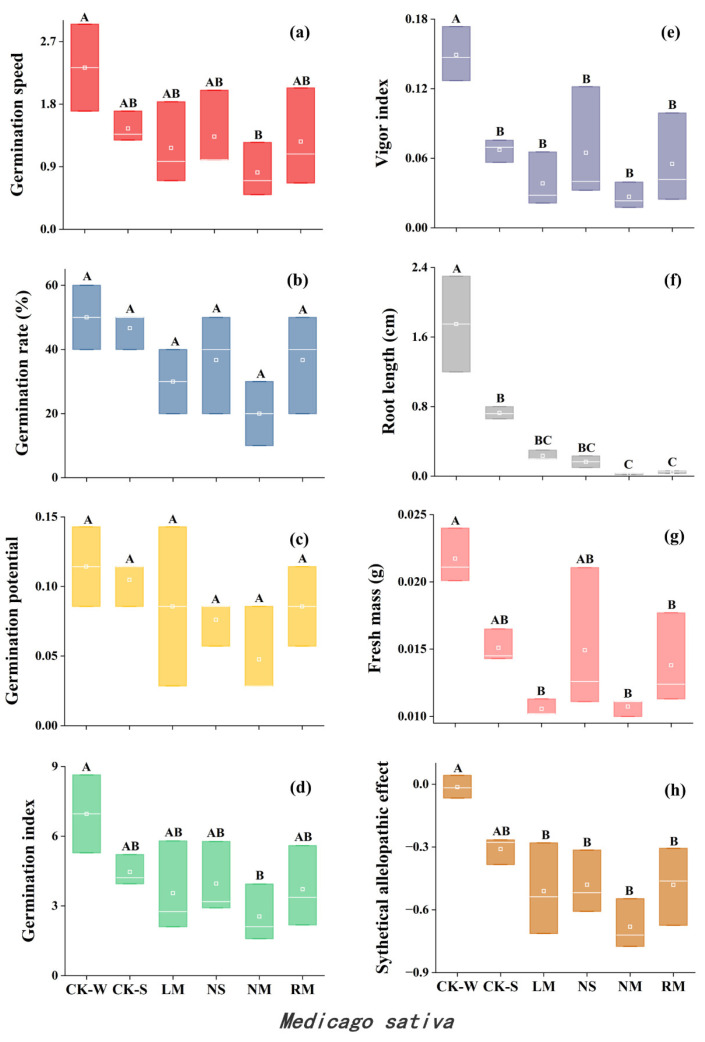
Allelopathic effects of aqueous extracts of *A. Philoxeroides* exerted by different sound treatments on germination speed (**a**), rate (**b**), potential (**c**), index (**d**) vigor index (**e**) root length (**f**), fresh mass (**g**) and synthetic allelopathic effect (**h**) of *Medicago sativa*. Note: Bars labeled with different letters represent significantly different (*p* < 0.05; one-way ANOVA), bars represent standard errors; CK-W: control of distilled water, CK-S: control without sound treatment, LM: light music, NS: natural sound, NM: noise sound, and RM: rock music.

Compared with the water control, aqueous extracts of invasive alligator weed significantly inhibited seed germination (germination speed, germination index, vigor index, and synthetic allelopathic effect) and seedling growth (root length and fresh mass) in *M. sativa* (Figure 2 and Figure 3, *p* < 0.05), although no significant effects were observed on germination rate or germination potential. Specifically, while neither germination rate nor germination potential differed among the six treatments (Figure 3, *p* > 0.05), root length suppression in *M. sativa* increased with the sound intensity applied to the source plants (Figure 3, *p* < 0.05). In contrast, germination speed, germination index, vigor index, synthetic allelopathic effect, and fresh mass did not vary significantly among extracts from plants exposed to the five sound treatments (Figure 3, *p* > 0.05).

### 3.3. Allelopathic Effects on L. sativa

**Figure 4:**
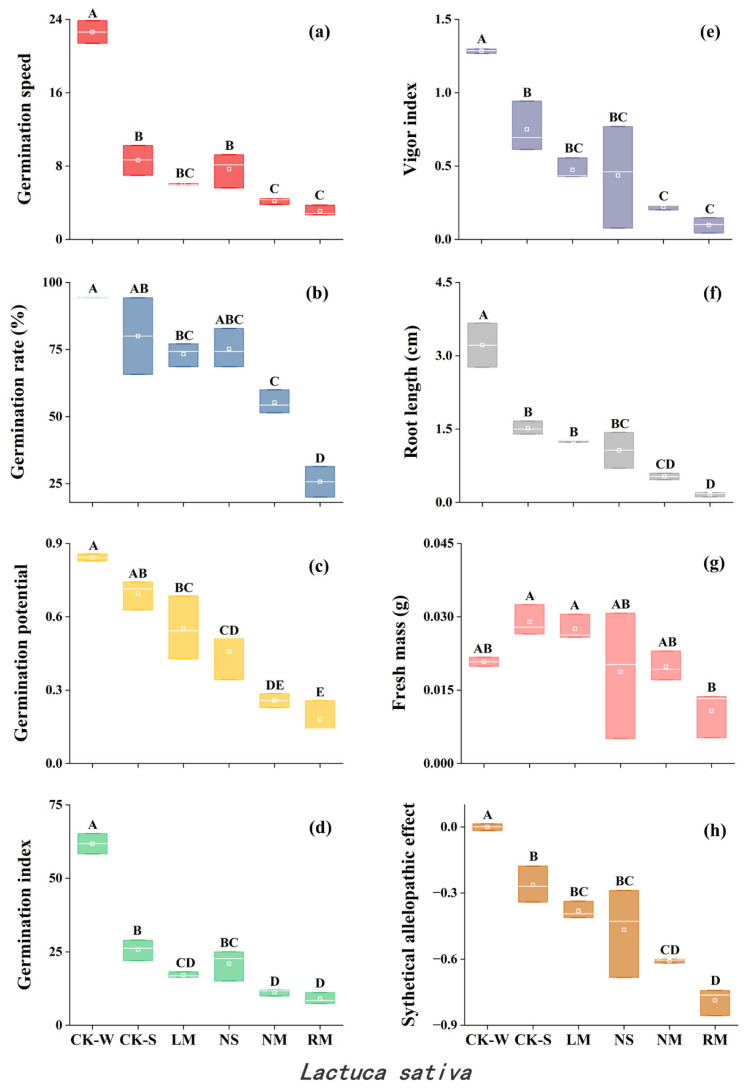
Allelopathic effects of aqueous extracts of *A. philoxeroides* exerted by different sound treatments on germination speed (**a**), rate (**b**), potential (**c**), index (**d**) vigor index (**e**) root length (**f**), fresh mass (**g**) and synthetic allelopathic effect (**h**) of *Lactuca sativa*. Note: Bars labeled with different letters represent significantly different (*p* < 0.05; one-way ANOVA), bars represent standard errors; CK-W: control of distilled water, CK-S: control without sound treatment, LM: light music, NS: natural sound, NM: noise sound, and RM: rock music.

Similarly, compared with the water control, aqueous extracts of invasive alligator weed significantly inhibited seed germination (germination speed, germination rate, germination potential, germination index, vigor index, and synthetic allelopathic effect) and seedling growth (except root length) in *L. sativa* (Figure 2 and Figure 4, *p* < 0.05). While fresh mass of *L. sativa* did not differ among the six treatments (Figure 4, *p* > 0.05), the suppression of root length, germination speed, germination rate, germination potential, germination index, vigor index, and synthetic allelopathic effect increased with the sound intensity applied to the donor plants (Figure 4, *p* < 0.05).

### 3.4. Two-Way ANOVA Results

The measured indices differed significantly between the two species and among the six sound treatments (Table 1, *p* < 0.05), except for the synthetic allelopathic effect, which did not vary significantly between species (*p* > 0.05). Species and sound treatment together explained > 70% of the total variance in all indices except fresh mass, for which the explained variance was 44.55%. Significant species × sound-treatment interactions were also detected for all indices (Table 1, *p* < 0.05), except for the synthetic allelopathic effect (*p* > 0.05). These interactions accounted for >16% of the total variance, except for germination rate (10.05%) and root length (5.14%).

## 4. Discussion

Our findings demonstrate that long-term exposure to sound, even at low intensity, inhibits the growth of *A. philoxeroides* while enhancing its physiological responses, with the magnitude of these effects increasing with sound intensity. However, *A. philoxeroides* did not clearly distinguish between rhythmic and non-rhythmic sound, despite the observed increase in allelopathic intensity with higher sound levels.

### 4.1. Plant Growth Inhibition

Contrary to previous findings that short-term exposure to low-intensity (60–100 dB) sound generally promotes plant growth and performance [21,23,24,26,32,33], our study revealed that long-term sound exposure, even at low intensity, significantly inhibited the growth of alligator weed, including its plant mass, SPAD, and relative growth rate (Figure 1). The results indicate that plant growth can also be inhibited by low-intensity sound if the exposure duration is sufficiently long, as sound represents an abiotic physical stress for plants [1,35]. The previously reported growth promotion under short-term, low-intensity sound exposure may occur because the imposed acoustic stress is insufficient to cause negative effects, rather, it acts as a stimulus that triggers molecular adjustments, thereby enhancing plant tolerance and promoting growth under acoustic and other adverse conditions [14,29]. Consistent with this, plant growth is generally promoted by low-intensity sound [21,34,47,48,49] but inhibited by high-intensity sound [19,32,37], which can be attributed to the greater acoustic energy or pressure carried by high-intensity sound [21,27]. Consistent with the upregulation of antioxidant defenses (total phenols, MDA, and SOD) in *A. philoxeroides* at high sound intensities, the increased stress likely stimulated higher allelochemical secretion, thereby amplifying the allelopathic effects [27,46]. Accordingly, sound effects on plant growth are functions of both intensity and duration. Consequently, to maintain plant fitness and yield, especially in crops, prolonged exposure to sound should be avoided even when the sound intensity is low.

Our results agreed well with former research, which reports that sound-induced growth inhibition becomes more pronounced as frequency [32] or intensity increases [20,50]; the growth of alligator weed in the present study also declined with increasing sound intensity (Figure 1). These findings suggest that sound with higher intensity and wavelength exerts stronger stress and causes more severe physiological damage to plants, likely because it carries greater acoustic energy or pressure, as noted earlier.

### 4.2. Physiological Responses

As one of the most sensitive systems in responding to biotic and environmental stresses, plant physiology typically reacts earliest to acoustic stress, whereas morphological, vegetative, and reproductive changes require considerably more time to develop [51]. Many plants have been found to activate their own innate immune responses as a systemic defense following exposure to sound stress [10,14]. In alignment with this, the enzyme activities of SOD and MDA in invasive alligator weed were also upregulated, indicating their important roles in mitigating sound stress [10,14,32]. Notably, certain secondary metabolites, such as the total phenols measured in this study, are induced by sound waves and contribute to antioxidant defense mechanisms in plants [19,52]. Furthermore, even under conditions where growth and physiological fitness are enhanced, increases in secondary metabolites and enzyme activities can be stimulated to counteract reactive oxygen species generated by low-intensity sound or rhythmic acoustic waves [19,52]. Together with our findings, these results indicate that plants experience physiological stress even at low sound intensities or with short-wavelength exposure, despite apparent benefits in other external traits, verifying the stress nature of sound waves [1,27]. For instance, arugula exhibits a significant reduction in biospeckle activity within minutes of exposure to various sound stimuli [53]. This suggests that plants may obtain benefits from low-intensity sound only when their defense systems are capable of mitigating its adverse effects. However, beyond a certain threshold, these protective mechanisms become insufficient. Therefore, optimizing acoustic treatments to maximize plant benefits while minimizing stress-induced damage represents a key challenge for the future application of sound technology in horticulture and agriculture [29,31].

Our findings imply that long-term exposure to low-intensity sound suppresses *A. philoxeroides* growth and stimulates its antioxidant defenses. However, the potential interference from background noise cannot be entirely ruled out. Despite the limited intensity and frequency of this noise, it may have influenced the sound treatments. Therefore, further replication of this study is warranted to validate these findings before drawing definitive conclusions.

### 4.3. Allelopathic Effects

Following sound exposure, the aqueous extract of invasive alligator weed exhibits enhanced allelopathic effects on the two target species compared with the control (no sound exposure), with allelopathic activity increasing proportionally with sound intensity (Figure 2, Figure 3 and Figure 4, Table 1). These observations align with previously noted growth and physiological responses of alligator weed to acoustic stimuli. Specifically, plant growth inhibition, enzymatic activity, and antioxidant compound levels are all elevated in response to increasing sound intensity. Correspondingly, the heightened allelopathic effect can be attributed to increased secretion of secondary metabolites, such as total phenols in this study, under stronger acoustic stress (Figure 1). Typically, elevated concentrations of secondary metabolites are linked to a pronounced upregulation of transcription factor genes [19]. Consequently, the inhibition of seed germination and seedling growth in the two recipient species also intensifies with the sound intensity applied to the donor alligator weed. Nevertheless, the two target species, and even different measured attributes (seed germination vs. seedling growth) within the same species, respond differentially to the aqueous extract, reflecting variation in trait susceptibility and inherent allelopathic resistance between species [42,46,54]. The greater susceptibility of *L. sativa* to the aqueous extract of alligator weed, compared with *M. sativa*, may be attributed to differences in their evolutionary histories [42,46]. However, the uniform germination conditions employed in this study may limit the interpretation of our results, as the two species exhibit distinct germination requirements.

### 4.4. Distinguishment of Sound Rhythm

Based on the present findings, we conclude that invasive alligator weed does not clearly distinguish between rhythmic and non-rhythmic sound, as neither its growth inhibition nor physiological responses differ significantly between the two sound types at either low or high intensities, although the classification of sound into rhythmic versus arrhythmic categories in this study may be somewhat arbitrary (Figure 1). This suggests that plants are more sensitive to sound intensity than to acoustic rhythm, and that their perception of sound fundamentally differs from that of humans and animals although plants can respond distinctly to different vibrational sound patterns [19]. Unlike animals, which possess specialized auditory organs, plants detect sound primarily through mechanical channels by their touch receptors [15] and lack dedicated sound-sensory structures [5,9,10,11,12]. Consequently, while plant sound perception is well documented [12,13,14], their capacity to discriminate rhythmic patterns appears limited. Moreover, acoustic responses vary considerably among plant species and are influenced by factors such as developmental stage, vibration characteristics, application time, exposure duration, and treatment regime [19,32,53,55]. It is important to note that the observed physiological indices are indirect measures of acoustic rhythm perception. Subsequent investigations should aim to elucidate the underlying mechanisms through sound-specific indicators, specifically mechanosensitive ion channels and cell wall dynamics [56,57]. Given that only a single species was examined here, further studies are needed to clarify plants’ ability to distinguish sound rhythms before general conclusions can be drawn or practical applications developed.

## 5. Conclusions

Our results demonstrate that prolonged exposure to sound, even at low intensity, inhibits the growth of *A. philoxeroides* while activating its defense mechanisms, with the magnitude of these responses increasing with sound intensity. However, the plant showed no clear distinction between rhythmic and non-rhythmic sound, despite exhibiting enhanced allelopathic effects at higher intensities. This study highlights the limited ability of plants to discriminate sound rhythms and underscores the negative impacts of prolonged low-intensity sound on plant performance. Therefore, the application of sound-based treatments in horticulture and agriculture must carefully balance potential benefits against possible adverse effects on plants, such as short-term exposure to low-intensity sound, particularly light music.

## Figures and Tables

**Table 1:** Two-way ANOVA results and percentage (%) of explained variance of allelopathic effects for the eight indices of two target species in response to extracts of *Alternanthera philoxeroides* exerted by different sound treatments. Note: Values of *p* < 0.05 are in bold.

Indices	Source	Percentage (%)	F	*p*
Germination speed	Species (S)	38.01	586.59	**<0.001**
	Sound (M)	34.24	105.679	**<0.001**
	S × M	26.20	80.88	**<0.001**
	Error	1.56		
Germination rate	Species (S)	75.48	907.61	**<0.001**
	Sound (M)	12.47	29.98	**<0.001**
	S × M	10.05	24.18	**<0.001**
	Error	2.00		
Germination potential	Species (S)	59.14	442.50	**<0.001**
	Sound (M)	21.50	32.01	**<0.001**
	S × M	16.11	24.47	**<0.001**
	Error	3.24		
Germination index	Species (S)	38.59	612.28	**<0.001**
	Sound (M)	34.35	109.00	**<0.001**
	S × M	25.55	81.09	**<0.001**
	Error	1.51		
Vigor index	Species (S)	39.89	586.59	**<0.001**
	Sound (M)	31.63	105.67	**<0.001**
	S × M	22.18	80.88	**<0.001**
	Error	6.37		
Root length	Species (S)	18.79	102.39	**<0.001**
	Sound (M)	71.66	78.09	**<0.001**
	S × M	5.14	5.61	**<0.001**
	Error	4.40		
Fresh mass	Species (S)	21.67	18.06	**<0.001**
	Sound (M)	22.88	3.81	**0.01**
	S × M	26.65	4.44	**0.01**
	Error	28.80		
Synthetical allelopathic effect	Species (S)	0.01	0.02	0.905
	Sound (M)	76.41	23.33	**<0.001**
	S × M	7.85	2.40	0.067
	Error	15.72		

## Data Availability

The original contributions presented in this study are included in the article. Further inquiries can be directed to the corresponding authors.

## Supplementary Materials

The following supporting information can be downloaded at: https://www.mdpi.com/article/10.3390/plants15111678/s1.

## Abbreviations

CK-W	control (distilled water)
CK-S	control (extract solution of *A. philoxeroides* without sound treatment)
LM	light music
NS	natural sound
NM	noise sound
RM	rock music
Hz	hertz
dB	decibel
RGR	relative growth rate
MDA	malondialdehyde
SOD	superoxide dismutase
GS	germination speed
GR	germination rate
GP	germination potential
GI	germination index
RL	root length
FM	fresh mass
VI	vigor index
